# Mtor inhibition by INK128 extends functions of the ovary reconstituted from germline stem cells in aging and premature aging mice

**DOI:** 10.1111/acel.13304

**Published:** 2021-01-14

**Authors:** Dai Heng, Xiaoyan Sheng, Chenglei Tian, Jie Li, Linlin Liu, Mo Gou, Lin Liu

**Affiliations:** ^1^ State Key Laboratory of Medicinal Chemical Biology Nankai University Tianjin China; ^2^ Department of Cell Biology and Genetics College of Life Sciences Nankai University Tianjin China; ^3^ Animal Resources Center Nankai University Tianjin China

**Keywords:** aging, Hormone, INK128, primordial germ cells, rapamycin, reconstituted ovary, stem cell transplantation

## Abstract

Stem cell transplantation has been generally considered as promising therapeutics in preserving or recovering functions of lost, damaged, or aging tissues. Transplantation of primordial germ cells (PGCs) or oogonia stem cells (OSCs) can reconstitute ovarian functions that yet sustain for only short period of time, limiting potential application of stem cells in preservation of fertility and endocrine function. Here, we show that mTOR inhibition by INK128 extends the follicular and endocrine functions of the reconstituted ovaries in aging and premature aging mice following transplantation of PGCs/OSCs. Follicular development and endocrine functions of the reconstituted ovaries by transplanting PGCs into kidney capsule of the recipient mice were maintained by INK128 treatment for more than 12 weeks, in contrast to the controls for only about 4 weeks without receiving the mTOR inhibitors. Comparatively, rapamycin also can prolong the ovarian functions but for limited time. Furthermore, our data reveal that INK128 promotes mitochondrial function in addition to its known function in suppression of immune response and inflammation. Taken together, germline stem cell transplantation in combination with mTOR inhibition by INK128 improves and extends the reconstituted ovarian and endocrine functions in reproductive aging and premature aging mice.

## Graphical abstract

In this manuscript, Heng et al. report that mTOR inhibition by INK128 extends functions of ovarian reconstituted from transplantation of primordial germ cells/oogonia stem cells using various mouse models such as young mouse model, natural aging, premature aging mouse by knockout of telomerase, and immunodeficient mice. Moreover, INK128 greatly elevates mitochondria functions and suppresses inflammation and immunoresponses in the reconstituted ovaries.

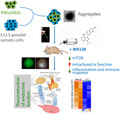

## 1 INTRODUCTION

The ovary maintains the normal function of the female reproductive system and hormone secretion. With the maternal age, periodic ovulation, follicular atresia, and apoptosis cause the number of follicles to drop sharply, leading to ovarian senescence or aging (Broekmans et al., 2007; Djahanbakhch et al., 2007; Hansen et al., 2005). Female fertility declines from about 35 years of age, and woman enters menopause at about 50 years of age, disrupting endocrine function (te Velde & Pearson, 2002), and this can cause chronic diseases, such as coronary heart disease, osteoporosis, and endocrine disorders, greatly impacting women's physical and mental health (Chae & Derby, 2011; Kallen & Pal, 2011). Ovarian aging can cause disturbances in the regulation of hormone secretion in the hypothalamus–pituitary–ovarian axis (Danilovich et al., 2002), leading to an increase in follicle‐stimulating hormone (FSH) levels and a decline in the levels of FSH‐negative regulators inhibin and anti‐Müllerian hormone (AMH). Elevated FSH increases the follicular atresia rate and accelerates follicular wear, which affects the secretion of estrogen in the body and eventually causes menopause (Lawson et al., 2003). Menopause prior to the age of 40 is considered pathological ovarian aging, or premature ovarian failure (POF) (Conway, 2010; Jin et al., 2012), and the reduced follicles also disrupt sex hormone (Qin et al., 2014; Titus et al., 2013; Zhao et al., 2013). Lack of germline stem cells (GSCs) and neo‐oogenesis in the adult ovaries revealed by lineage tracking previously (Lei & Spradling, 2013; Zhang et al., 2012) is consistent with the notion that finite egg production and follicle reserve are not replenished in adult women and in female mice (Kerr et al., 2012; Telfer & Albertini, 2012; Tingen et al., 2009). Hence, the depletion of oocytes and follicular reserve leads to ovarian dysfunction with age and also POF.

Primordial germ cells (PGCs) are the precursor cells of oogonia or eggs and sperm (Gunesdogan & Surani, 2016; Saitou & Miyauchi, 2016; Surani et al., 2007). PGCs undergo mitotic cell division until E12.5 when they immediately enter meiosis by E13.5 facilitated by retinoic acid‐mediated Stra8 stimulation (Endo et al., 2015, 2019; Lei & Spradling, 2016; Lin et al., 2008; Saitou & Miyauchi, 2016). Because PGCs can undergo self‐renewal and differentiation to meiocytes, these cells also were functionally comparable to so‐called GSCs or oogonia stem cells (OSCs) (Sheng et al., 2019; Zeng et al., 2017). It has been hoped that germline stem cell transplantation can reconstitute ovarian oocyte and follicular development and endocrine function. Indeed, PGCs following aggregation with embryonic gonadal somatic cells can be induced to undergo meiosis and follicular development and form oocytes by transplanting the recombinant gonads into the kidney cysts of mice (Chen et al., 2013; Hayama et al., 2014; Matoba & Ogura, 2011; Qing et al., 2008; Shen et al., 2006), but surprisingly the reconstituted ovaries can only sustain their functions for only about 4 weeks or so (Zeng et al., 2017). Despite several potential factors, premature activation of follicle development presumably contributes significantly to the depletion of the transplanted germ cells, and this likely involves in mTOR signaling.

It has been shown that the kinase mammalian/mechanistic target of rapamycin (mTOR) signaling is increased with aging and inhibition of mTOR partly reverses aging and associated dysfunction (Field & Adams, 2017; Johnson et al., 2013; Liu & Sabatini, 2020; Newgard & Sharpless, 2013; Tang et al., 2019). As a drug that inhibits the mTOR pathway, rapamycin fed late or mid in life extends life span of both male and female mice (Harrison et al., 2009). Excitingly, rapamycin not only extends life span, but it also prevents or delays the onset of age‐related diseases such as cancer and Alzheimer's disease, rejuvenates the aging mouse heart, and ameliorates age‐related cognitive decline (Kennedy & Lamming, 2016; Longo et al., 2015). Interestingly, the activation of the mTOR pathway is important for the activation of primitive follicles (Adhikari & Liu, 2009; Adhikari et al., 2010, 2013; Zhang & Liu, 2015). Indeed, rapamycin treatment delays or inhibits rapid activation of the primordial follicles and follicular development (Adhikari et al., 2013; Zhang et al., 2013, 2017). Moreover, mTOR inhibition has been shown effective to preserve ovarian follicular reserve in mice exposed to chemotherapy (Goldman et al., 2017). The clinically mTOR kinase inhibitor, such as the clinically approved drug that inhibits mTORC1 everolimus (RAD001) or experimental drug INK128 that inhibits mTORC1/2, can protect ovarian function and preserve normal fertility (Goldman et al., 2017).

Although rapamycin has shown a potential for preserving the ovarian follicle pool and preventing premature ovarian failure, the application is limited because of its detrimental effects on follicular development and ovulation during long‐term treatment (Dou et al., 2017). Short‐term rapamycin treatment for 2 weeks appears to preserve primordial follicles, increases oocyte quality, and improves the ovarian microenvironment, prolonging ovarian life span (Dou et al., 2017). Intriguingly, mTOR pathway plays important roles in subsequent development of primordial and growing oocytes, as complete deletion of *Mtor* increases DNA damage, reduces oocyte quality, and causes infertility (Guo et al., 2018). Nevertheless, as the ovarian reserve is set at birth, the depletion of germ cells and follicles eventually leads to ovarian senescence without replenishment of GSCs (Kerr et al., 2012; Lei & Spradling, 2013; Telfer & Albertini, 2012; Tingen et al., 2009; Zhang et al., 2012). It remains elusive whether the transplantation of germline stem cells can reconstitute ovarian reserve and recover endocrine functions. Here, we show that the appropriate inhibition of mTOR can postpone follicular activation and development and extend the follicle reserve and endocrine functions of the ovaries reconstituted from transplantation of PGCs/GSCs in reproductive aging and premature aging mice.

## 2 RESULTS

### 2.1 Rapamycin delays folliculogenesis in the reconstituted ovaries following germline stem cell transplantation

**FIGURE 1:**
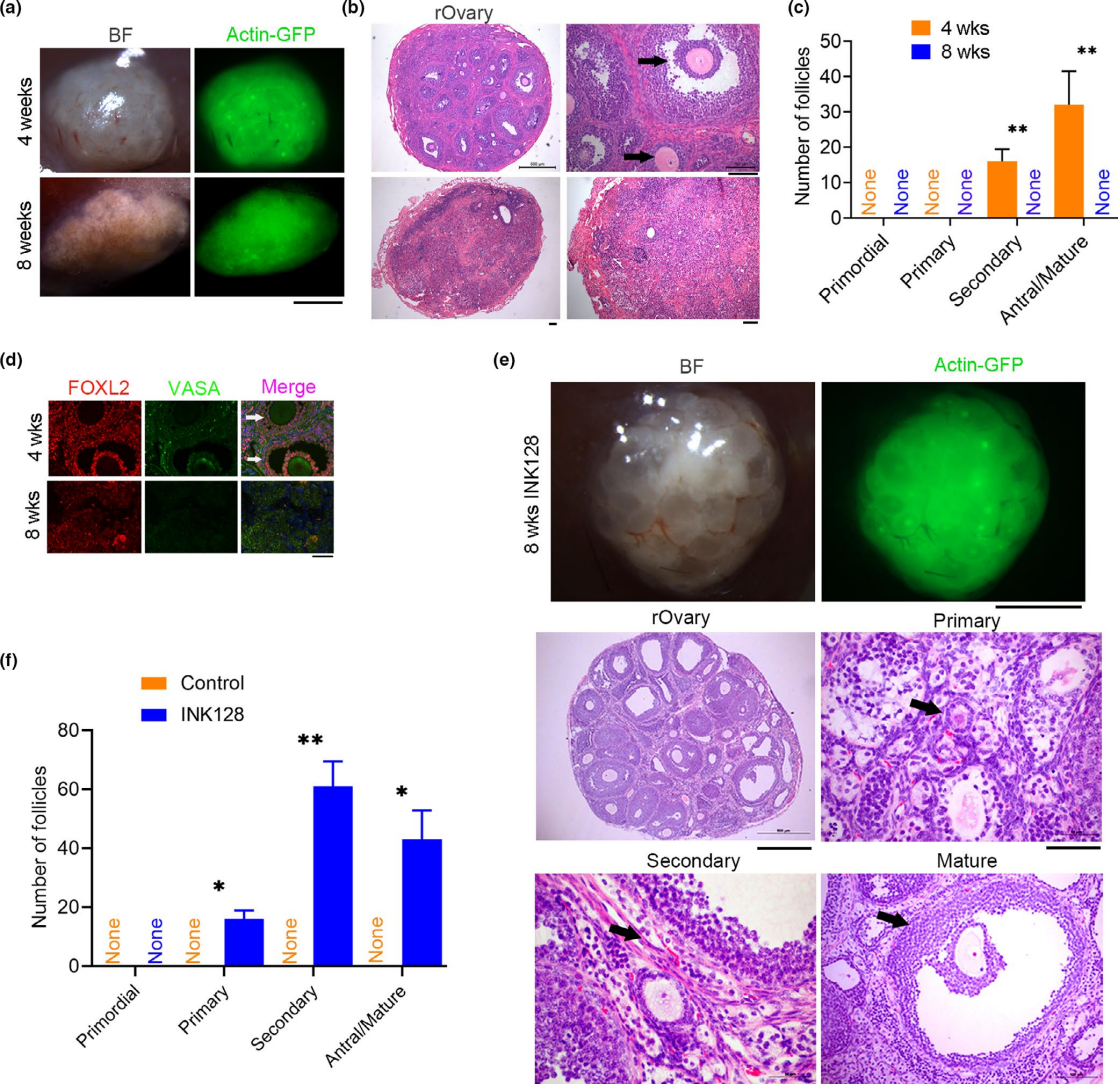
INK128 maintains follicular development in the reconstituted ovaries (rOvary) of young recipient C57BL/6 mice. (a) Morphology of the rOvary four or 8 weeks following transplantation of PGCs aggregated with E12.5 gonadal somatic cells into young recipient C57BL/6 mice (2–3 months old, an average of 10 weeks old) treated with or without INK128 (control). BF, microscopic image under bright field; actin‐GFP indicates donor cell sources from mice carrying actin‐GFP. Scale bar = 1 mm. (b) Histology of rOvary sections by H&E staining indicating various follicles 4 weeks, but the absence of follicles 8 weeks following transplantation. The black arrows refer to secondary and mature follicles. Scale bar = 100 μm. (c) Number of follicles at various developmental stages in the rOvary four or 8 weeks after transplantation of PGCs. *n* = 3. (d) Immunofluorescence of FOXL2 indicating granulosa cells in the follicles and VASA to label oocytes. Scale bar = 50 μm. (e) Follicles maintained in the rOvary of recipient mice by treatment with INK128 for 8 weeks. Upper panel, morphology of rOvary; scale bar = 1 mm. Bottom panel, histology of rOvary sections by H&E staining displaying various follicles. Scale bar = 500 μm. Shown also are primary, secondary, and mature follicles indicated by black arrows at higher magnifications (scale bar = 100 μm). (f) Number of follicles at various developmental stages in the rOvary 8 weeks after transplantation of PGCs in the recipient mice treated with INK128, compared with control recipient mice without INK128 treatment. Mean ± SEM. *p < 0.05, **p < 0.01

We isolated PGCs from transgenic mice that globally express actin‐GFP to distinguish donor cells from recipient mouse cells. Initially, we transplanted the aggregates of PGCs expressing actin‐GFP fluorescence with their E12.5 gonadal somatic cells into young C57BL/6 recipient mice and then collected reconstituted ovarian grafts (or named as rOvaries) 28 days/4 weeks or 2 months (8 weeks) following transplantation. A number of oocytes exhibiting GFP fluorescence were visible 28 days following transplantation (Figure 1a). Histology of rOvary sections by H&E staining revealed a number of secondary and antral or mature follicles 4 weeks following transplantation, but only corpus luteum and follicle‐free cavities and no follicles at 8 weeks (Figure 1b,c). Expression of germ cell marker protein VASA and granulosa cell marker protein FOXL2 in the transplants by immunofluorescence validated the follicular structures assembled from adult granulosa cells around oocytes in the 4‐week transplant but not in 8‐week transplant, although a small number of FOXL2‐positive cells were present (Figure 1d). Granulosa cells carrying GFP did not show GFP fluorescence in the routinely fixed wax‐embedded sections. Oocytes surrounded by granulosa cells were found at 4 weeks, and no oocytes present in the transplants at 8 weeks following transplantation of PGCs. These results further support previous findings that the reconstituted ovarian functions can only last for about 4 weeks following PGC transplantation (Zeng et al., 2017).

Next, we tested whether rapamycin delays follicular development in the rOvary by treating the recipient young C57BL/6 mice with or without (served as control) rapamycin in the drinking water. Rapamycin has been shown to inhibit target of rapamycin complex 1 (mTORC1) activation and thus rapid activation of the original ovarian follicles and follicular development (Adhikari et al., 2013; Zhang et al., 2013). A large number of oocytes were visible at 4 weeks in the rOvary following transplantation of PGCs, containing a number of primary follicles, and more secondary, antral, and mature follicles in the rapamycin‐treated mice than did control mice (Figure S1A–C). However, at 8 weeks following transplantation, the rOvaries contained more corpus luteum but almost no follicles (Figure S1A–C). These data suggest that normal ovulation and follicular atresia occur in the rOvary during 4–8 weeks, even though rapamycin noticeably increased number of oocytes and follicles by 4 weeks. Thus, rapamycin does not fully prevent immediate follicular activation and development for longer period of time in the rOvaries.

### 2.2 INK128 maintains folliculogenesis in the reconstituted ovaries following germline stem cell transplantation

The above unexpected results let us to test whether inhibition of TORC1/2 by INK128, which could be more effective in inhibiting mTOR signaling (Goldman et al., 2017), can be used to extend follicle development. Excitingly, the treatment of INK128 robustly maintained follicle development in the rOvary of young recipient C57BL/6 mice for at least 8 weeks. Donor germ cells/oocytes with GFP fluorescence were visible in the rOvary, containing a large number of primordial and primary follicles, but no secondary and mature follicles shown by H&E histology and by immunofluorescence of VASA and FOXL2 after feeding recipient mice with INK128 for 4 weeks (Figure S2A–C). These results demonstrated that INK128 is more effective in suppressing primary follicle activation, unlike rapamycin treatment. Moreover, continuous treatment of the recipient mice with INK128 for 8 weeks resulted in the development of oocytes and follicles at various stages expressing GFP fluorescence, many secondary and antral follicles, and also a number of primary follicles (Figure 1e,f). Hence, the inhibitory effect of INK128 on follicle activation delays follicular development of the rOvary for at least 8 weeks.

### 2.3 INK128 prolongs endocrine function of the reconstituted ovaries in aging mice

**FIGURE 2:**
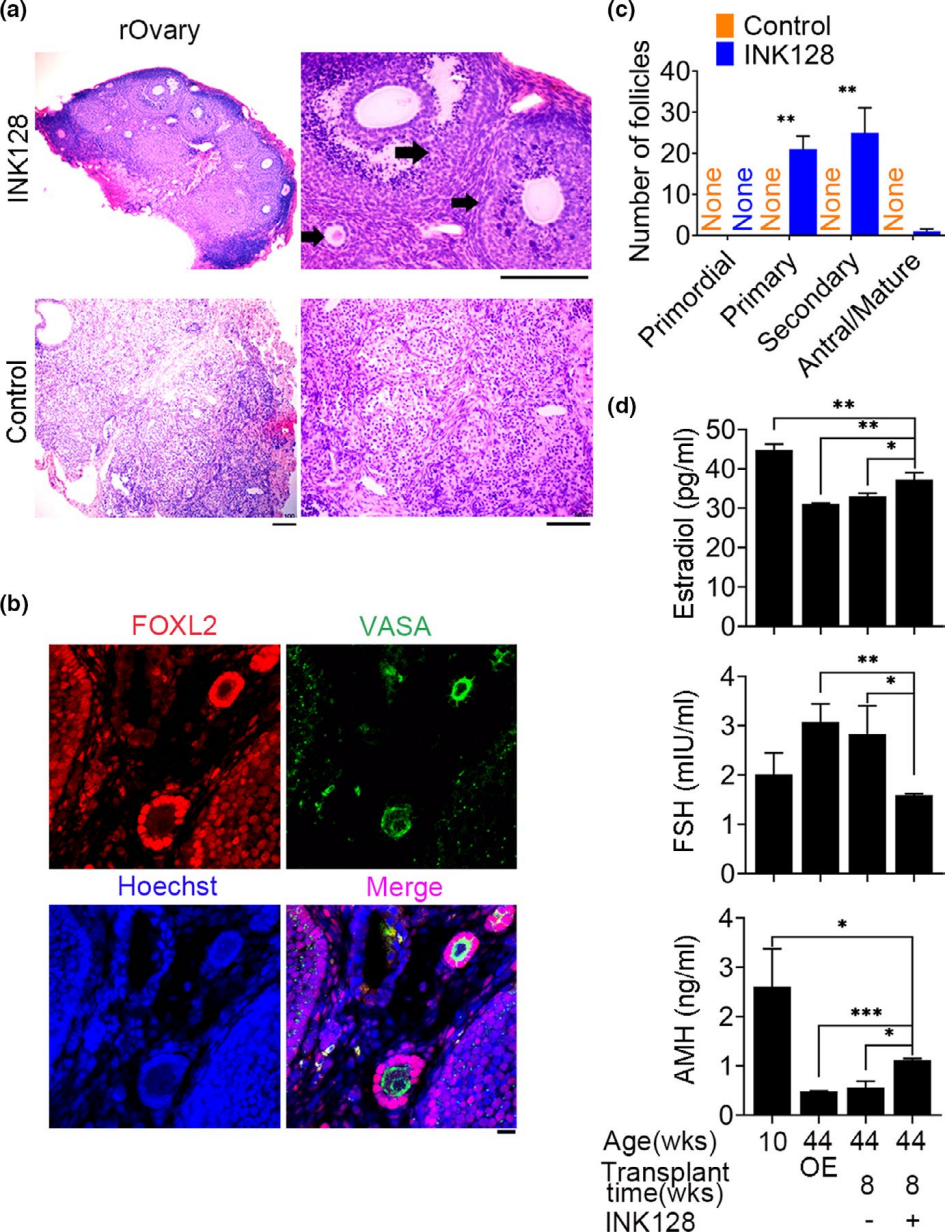
INK128 extends folliculogenesis in the rOvary of reproductive aging C57BL/6 recipient mice (36 weeks old) and endocrine functions. (a) Histology of actin‐GFP rOvary section by H&E staining displaying more primary and secondary follicles, and a small number of antral follicles (black arrows) in the rOvaries of old recipient mice following treatment for 8 weeks with INK128, but no follicle structure in the control mice without receiving INK128. Scale bar = 100 μm. (b) Immunofluorescence of germ cell marker VASA and granulosa cell marker FOXL2. Scale bar = 20 μm. (c) Number of follicles at various developmental stages in the rOvary 8 weeks after transplantation. *n* = 3. (d) Levels in the serum of follicle‐stimulating hormone (FSH), anti‐Müllerian hormone (AMH), and estradiol (E2) of recipient mice treated with (+) or without (−) INK128. C57BL/6 mice at the age of 10 weeks served as young control. C57BL/6 mice at 44 weeks old with a bilaterally ovariectomized (OE) served as negative controls of transplants. Each aggregate was transplanted beneath the kidney capsule of a bilaterally ovariectomized recipient mouse. Mean ± SEM. *N* ≥ 3. *p < 0.05, **p < 0.01, ***p < 0.001

The encouraging results from INK128 treatment experiments performed in young mice allowed us to test whether INK128 can actually rescue or extend age‐associated decline in endocrine functions by reconstructing ovaries from PGCs/GSCs. To do this, we treated with or without INK128 the old (8–10 months/an average of 36 weeks, reproductive aged) C57BL/6 recipient mice (Liu et al., 2013), following transplanting PGC aggregates with E12.5 gonadal somatic cells from the same actin‐GFP C57BL/6 fetus. At 8 weeks following transplantation of PGCs, the formed rOvaries exhibited oocyte and follicle development at primary, secondary, and antral stages in the old recipient mice treated with INK128, but not in those without receiving INK128 (Figure 2a,c). The oocytes and surrounding granulosa cells expressed germ cell marker VASA and granulosa cell marker FOXL2 by immunofluorescence (Figure 2a,b). Furthermore, FSH levels in the serum were reduced in the recipient mice containing rOvaries treated with INK128, compared with those without receiving INK128, and excitingly did not differ from those of young mice (Figure 2d). In contrast, AMH and estradiol levels were elevated in the recipient mice treated with INK128, compared with those without INK128 treatment. Elevated AMH and reduced FSH levels are commonly used as markers of high ovarian reserve and to indicate young ovarian functions too (Cohen et al., 2015; Xu et al., 2020; Zebitay et al., 2017). These data showed that the reconstructed ovary from transplanted germline stem cells retains the follicular development and endocrine function of old C57BL/6 recipient mouse for at least 8 weeks.

### 2.4 INK128 prolongs endocrine function of the reconstituted ovaries in premature aging mice

**FIGURE 3:**
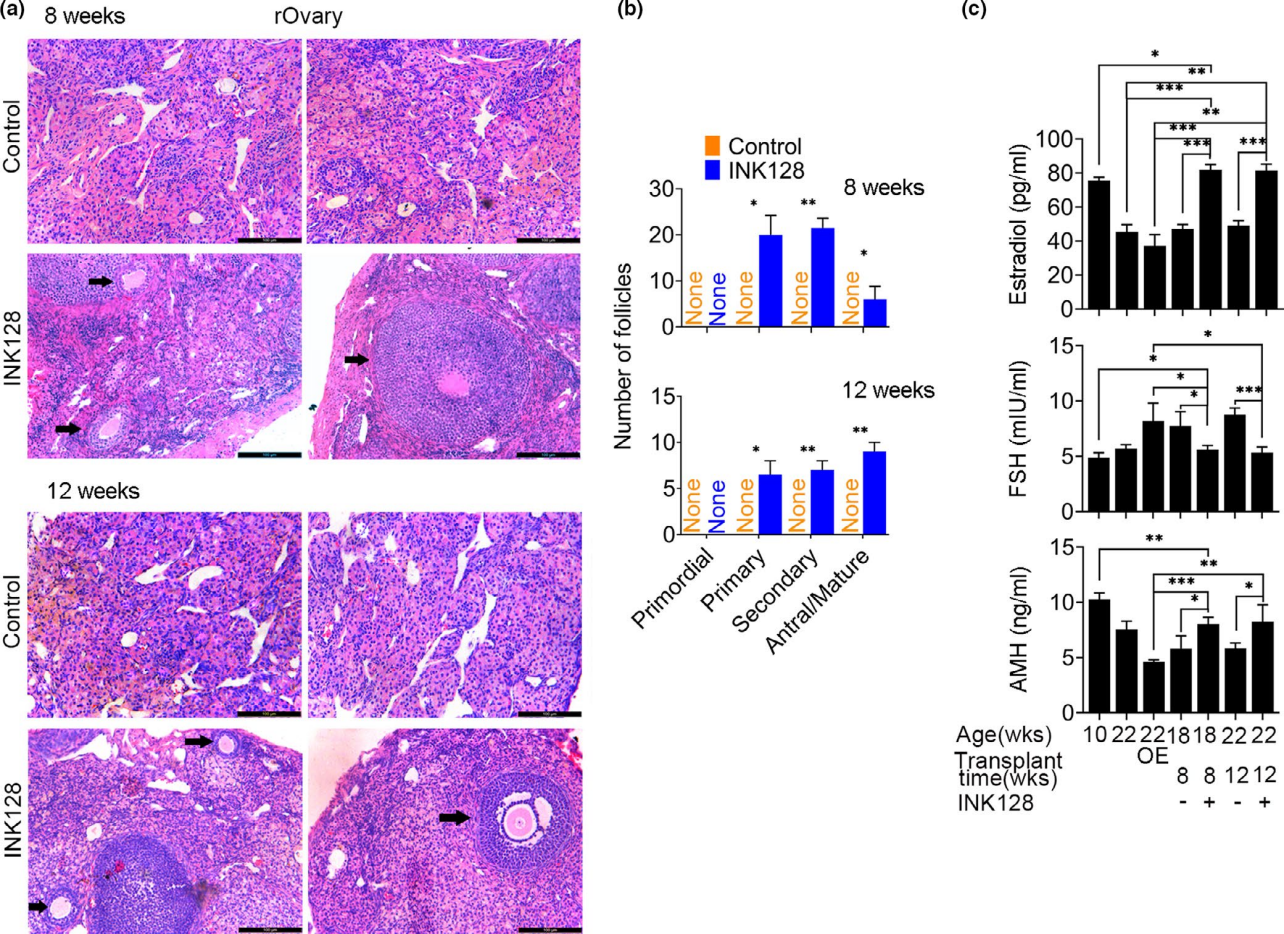
INK128 extends follicle development for 12 weeks in the rOvary of G2 *Terc* ^−/−^ mice. (a) Follicle development in the rOvary of G2 *Terc* ^−/−^ mice treated with or without INK128 for 8 or 12 weeks following transplantation of PGC aggregates. Representative images showing histology of rOvary sections by H&E staining. Scale bar = 100 μm. (b) Number of follicles at various developmental stages in the rOvary 8 or 12 weeks after transplantation. (c) Levels in the serum of follicle‐stimulating hormone (FSH), anti‐Müllerian hormone (AMH), and estradiol (E2) of recipient mice treated with (+) or without (−) INK128 following transplantation of PGCs for 8 or 12 weeks. G2 *Terc* ^−/−^ mice at the age of 10 weeks served as young control. G2 *Terc* ^−/−^ mice at 22 weeks old and those with a bilaterally ovariectomized (OE) served as negative controls of transplants. Each aggregate was transplanted beneath the kidney capsule of a bilaterally ovariectomized recipient mouse. Mean ± SEM. *n* ≥ 3. *p < 0.05, **p < 0.01, ***p < 0.001

To further test whether the formed rOvaries from transplanted PGCs could recover endocrine functions of premature aging mice, we performed similar experiments for an additional 1 month using 2nd‐generation telomerase‐deficient telomere shorted mice (G2 *Terc* ^−/−^) on C57BL/6 genetic background. The *Terc* ^−/−^ inbred C57BL/6 mice exhibited premature reproductive aging and infertility with increasing generations (Keefe, 2016; Keefe & Liu, 2009), earlier than the original hybrid telomerase‐deficient mice (Herrera et al., 1999), and G2 *Terc* ^−/−^ C57BL/6 females already had reduced fertility and declined ovarian functions at young age, so here employed as premature aging mouse models. At 8 weeks following transplantation of PGC aggregates from C57BL/6 carrying actin‐GFP fluorescence, the formed rOvaries contained primary, secondary, and antral follicles in the recipient G2 *Terc* ^−/−^ mice treated with INK128, but the transplants did not contain follicles in the recipient mice without INK128 treatment (Figure 3a, Figure S3a). About 3 months or 12 weeks following transplantation of PGCs, primary and secondary follicles and also mature follicles still were observed in the rOvaries of the G2 *Terc* ^−/−^ recipient mice treated with INK128 (Figure 3a,b, Figure S3B). Follicular structures assembled from granulosa cells indicated by FOXL2 around oocytes indicated by VASA were visible in the transplants after eight and 12 weeks (Figure S3C). Also, the levels of FSH, AMH, and estradiol in the serum of the recipients were assayed eight and 12 weeks after transplantation. The levels of FSH were lower, and levels of AMH and estradiol were higher in G2 *Terc* ^−/−^ mice at 2–3 months (young age) than those at 5–6 months. Compared with those of the bilaterally ovarian removal mice and recipient mice with transplants but without receiving INK128, FSH levels were decreased and the levels of estradiol and AMH elevated in the recipients with transplant‐formed rOvaries, treated with INK128. Notably, the estradiol levels were comparable to those of young mice at the age of 2–3 months (Figure 3c). Furthermore, we measured telomere length of reconstituted ovaries 4 weeks following transplantation of PGCs and compared with that of ovaries of G2 *Terc* ^−/−^ mice. Telomere length of reconstituted ovaries did not differ (*p* > 0.05) between INK128‐treated and control mice, but was evidently longer than that of G2 *Terc* ^−/−^ ovaries (Figure S3D). Moreover, genes associated with telomere maintenance and stabilization were upregulated by INK128 treatment (Figure S3E). These data indicate that INK128 extends the normal endocrine functions by maintaining follicle reserve and telomeres in the rOvaries for at least 12 weeks following PGC transplantation in the premature aging mice.

### 2.5 INK128 represses immune response and elevates mitochondria function

**FIGURE 4:**
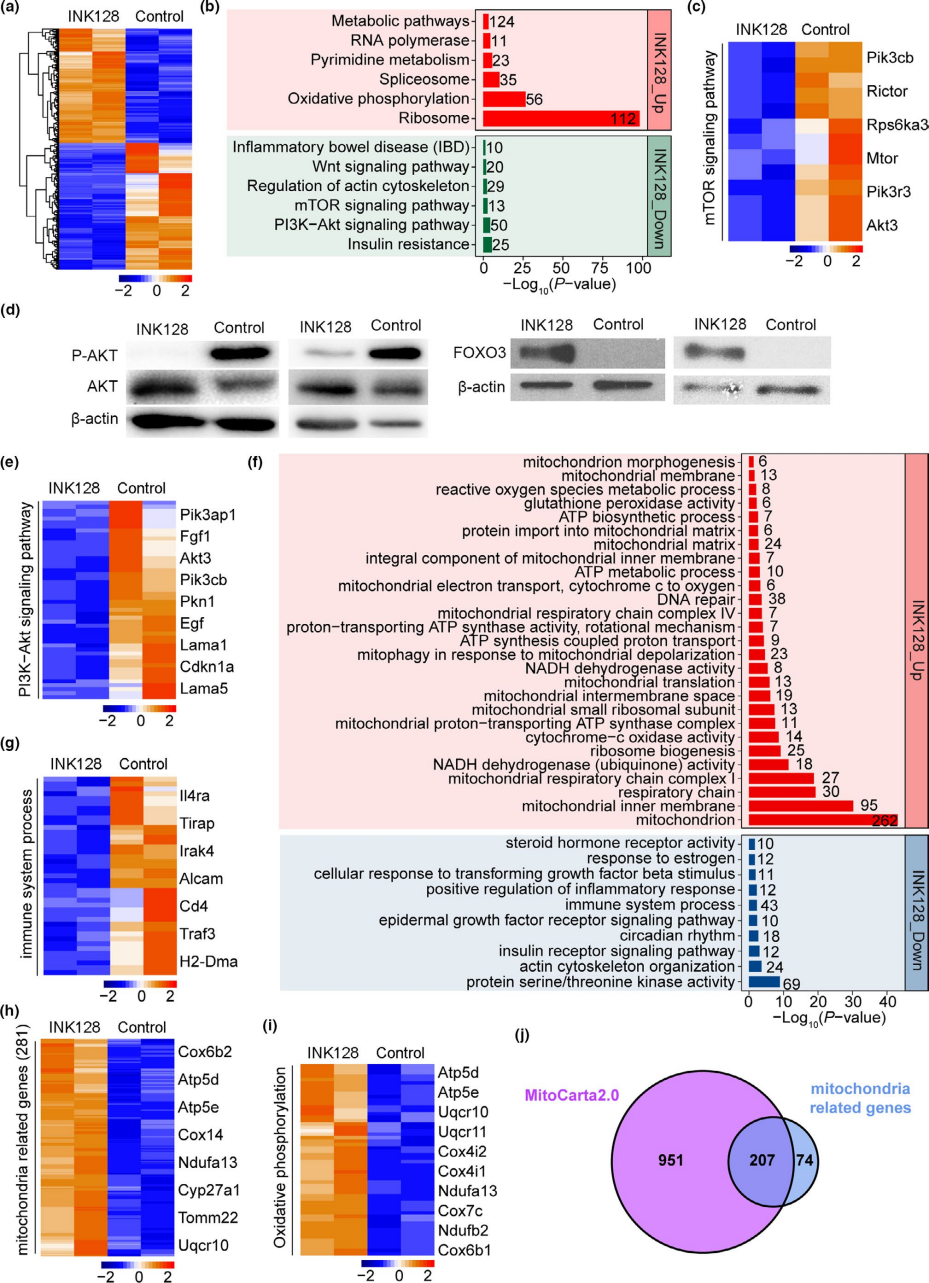
Effects of INK128 on the function of rOvaries through RNA‐seq analysis. (a) Heat map displaying differentially expressed genes (DEGs) in rOvaries of G2 *Terc* ^−/−^ mice between INK128 treatment and controls 4 weeks following transplantation. The color key from blue to red indicates the relative gene expression levels from low to high, respectively. (b) Representative signaling pathways enriched by DEGs in rOvaries between INK128 treatment and controls. Red and green bars represent signaling pathways enriched by upregulated and downregulated DEGs, respectively. (c) Heat map showing the expression levels of representative genes related to mTOR signaling pathway after INK128 treatment compared with controls. Key genes are listed on the right of the heat map. (d) Western blot analysis of protein expression of P‐AKT and FOXO3 in the rOvary from G2 *Terc* ^−/−^ mice treated with or without INK128 (control) for 4 weeks. (e) PI3 K‐Akt signaling pathway following INK128 treatment compared with controls. (f) Significantly enriched GO terms of DEGs in rOvaries between INK128 treatment and controls. Red and blue bars represent GO terms enriched by upregulated and downregulated DEGs, respectively. (g) Expression levels of genes associated with immune system process in rOvaries of INK128‐treated and controls, with several key genes listed on the right side. (h) Expression levels of mitochondria‐related genes in rOvaries of INK128‐treated and controls, with key genes listed on the right. (i) Expression levels of genes associated with oxidative phosphorylation in INK128‐treated and controls. (j) Venn diagram illustrating genes shared between MitoCarta2.0 dataset and mitochondrial‐related genes revealed in our data analysis

To understand the effects of INK128 on the functions of rOvaries at molecular levels, we performed RNA‐seq analysis of the rOvaries collected from G2 *Terc* ^−/−^ recipient mice treated with or without INK128 served as control. The rOvaries were harvested 4 weeks after transplantation. Firstly, we identified differentially expressed genes (DEGs) in the comparison between INK128‐treated mice and controls, showing 1,648 upregulated and 1,817 downregulated genes by INK128 (Figure 4a). While the metabolism and oxidative phosphorylation were upregulated, Wnt, mTOR, and PI3 K‐Akt signaling pathways downregulated in the rOvaries of the recipient mice by treatment with INK128, compared with controls without INK128 (Figure 4b–e), consistent with known functions of INK128 as mTOR inhibitor in the inhibition of these pathways. By Western blot, INK128 decreased Akt pathway activation as evidenced by noticeable reduction in phosphorylation of S473 Akt compared with untreated controls (Figure 4d). Meanwhile, the protein expression of Foxo3 downstream effector of mTOR was increased through treatment with INK128.

In addition, GO analysis revealed that downregulated DEGs were enriched for functions related to immunity and inflammation (Figure 4f,g), also supporting the INK128 as mTOR inhibitor, which was originally discovered to exhibit immunosuppressive activity (review (Johnson et al., 2013)). Remarkably, upregulated DEGs by INK128 were enriched for functions and components associated with mitochondria (Figure 4f,h), coincided with the activation of oxidative phosphorylation (Figure 4i). Furthermore, these 281 mitochondrial‐related genes were well merged with MitoCarta2.0 dataset, which contained 1,158 mouse genes encoding mitochondrial proteins (Calvo et al., 2016; Pagliarini et al., 2008), and most of them (207) were shared (Figure 4j). Thus, INK128 suppresses immune response and promotes mitochondria functions.

### 2.6 INK128 similarly maintains folliculogenesis of the rOvary in immune‐deficient mice

To minimize potential immunorejection, the above transplantation was carried out by using the same genetic background, for example, PGCs from C57BL/6 to the kidney capsule of C57BL/6 recipient mice. To exclude the possibility of germ cell development and the follicles potentially reduced by immunorejection, we performed the transplantation experiments using immunodeficient NOD SCID recipient mice treated with or without INK128. Similar to those of C57BL/6 same genetic background transplants, large number of primordial and primary follicles, and only few secondary follicles, antral follicles, and mature follicles were found at 4 weeks in the rOvaries of the recipient NOD SCID mice treated with INK128 (Figure S4A). By 8 weeks, more secondary follicles and a few primary and antral follicles were found in the rOvaries of NOD SCID recipient mice treated with INK128 (Figure S4B). Follicles were further visualized by immunofluorescence of FOXL2‐stained granulosa cells and VASA in oocytes (Figure S4C and D). The total follicle numbers also approximated 40–50 in the rOvary of NOD SCID mice, comparable to those of allogeneic transplantation of PGCs at C57BL/6 genetic background. We also looked at follicle development 10 days following removal of INK128 from water after treatment for 8 weeks and observed certain number of follicles in the reconstituted ovaries (Figure S4E), suggesting recovery of follicular development without INK128. Fewer follicles were left, suggesting that follicles may have undergone ovulation or atresia, as anticipated. These results suggest that immunorejection, if any, unlikely hinders the germ cell and follicular development in the recipient mice following transplantation of PGCs at the same genetic background when mTOR inhibition is applied.

## 3 DISCUSSION

Stem cell aging and exhaustion are considered important drivers of organismal aging (Ren et al., 2017). Likewise, germ cell depletion with age also determines reproductive aging. We have shown that germline stem cell transplantation robustly regenerates oocyte and follicle development in young, reproductive aging, and premature aging mouse models. Excitingly, mTOR inhibition by INK128 greatly extends the follicle development and endocrine functions of the reconstituted ovary. Although our study focused on INK128 to extend the functions of germline stem cell transplants, the findings also may have implications in extending the efficacy of other somatic stem cells in regeneration and transplantation.

mTOR is an atypical serine / threonine kinase that is a metabolic sensor that regulates mRNA translation, cell growth, proliferation, autophagy signals, and survival (Laplante & Sabatini, 2012; Wullschleger et al., 2006). We show that mTOR inhibition can postpone the follicle development or activation in the reconstructed ovaries, further supporting role of mTOR in follicle activation shown previously (Adhikari & Liu, 2009; Adhikari et al., 2010, 2013). It appears that INK128 more effectively suppresses ovarian follicle activation than does rapamycin, and this could be related to inhibition of both mTORC1 and mTORC2 by INK128, whereas rapamycin only suppresses mTORC1 alone (Goldman et al., 2017; Johnson et al., 2013). Additionally, rapamycin has been found to alter mitochondria functions after longer‐term treatment, and melatonin administration reduces rapamycin‐associated toxicity to healthy cells (Shen et al., 2018). Also, the inhibition of mTOR by INK128 suppresses mitochondrial reactive oxygen species (ROS) production (Li et al., 2018), protecting mitochondrial and stem cell functions (Brand et al., 2016).

Rapamycin has been proven to have numerous negative side effects that would seem to preclude its wide‐scale use as an anti‐aging compound, but treatment dosage and time can affect the effectiveness and side effects of rapamycin (Kennedy & Lamming, 2016). In our preliminary experiments, short‐term or lower dosage treatment with rapamycin fails to suppress or delay primordial follicle activation and folliculogenesis, in contrast to INK128, which appears to be more effective and safer. After preliminary experiments on dosage dependence, we employed rapamycin at 40 mg/L or INK128 at 30 μM/L in water to treat the mice. Based on the evidence that certain number of primary and secondary follicles are observed in the reconstituted ovaries 12 weeks following transplantation of GSCs in the recipient mice treated with INK128 (Figure 3a), we anticipate that INK128 has protective effects on ovarian functions for longer term. Meanwhile, these mice treated with INK128 for 12 weeks appeared normal and healthy.

Suppression of immune response and elevated mitochondria function by INK128 revealed here could promote ovarian functions. Mitochondrial dysfunctions and inflammation are important aspects of aging processes (Ma, Sun, Geng, et al., 2020). Mitochondria function plays important role in ovarian follicular reserve and mitochondria dysfunctions, and aberrant metabolisms are implicated in ovarian aging and infertility (Cecchino et al., 2018; Seidler & Moley, 2015; Yang et al., 2020; Zhang et al., 2019). Disorders of aging are frequently associated with mitochondrial dysfunction, as are impaired oogenesis and embryogenesis (Kasapoglu et al., 2020). Enhanced mitochondrial functions could make one of important contributions to reverse aging and ovarian aging too (May‐Panloup et al., 2016). Mitochondria dysfunction in stem cells is implicated in aging, and improving mitochondrial and stem cell function by NAD(+) repletion enhances life span in mice (Zhang et al., 2016, 2018). INK128 promotes NADH dehydrogenase activity, mitochondria functions, and metabolisms, and these can contribute to extended ovarian functions with age. Supplementing INK128 through diet or administration can prolong reproductive life span and improve the multifaceted effects of reproductive aging on the body.

Immunoactivity or inflammation response also can cause follicle activation and deplete follicles, leading to POF (Bukovsky & Caudle, 2008, 2012). Reducing inflammation by knockout of interleukin 1 elevates serum levels of anti‐Mullerian hormone (AMH) and ovarian reserve, and also prolongs ovarian life span in mice (Uri‐Belapolsky et al., 2014). An original study revealed that the activation of PI3 K signaling due to lacking PTEN leads to the activation of entire primordial follicle pool and thus depletion of follicles, causing POF (Reddy et al., 2008). Consistently, our data indicated that mTOR inhibitor INK128 downregulates activity of P‐AKT/PI3 K and upregulates expression of FOXO3. FOXO signaling and balanced mitochondrial function modulate longevity and extend life span (Mouchiroud et al., 2013). Hence, INK128 regulates the follicles dormant or awaken through AKT/FOXO3 pathway in the rOvaries and also likely can postpone the processes of POF.

Increased inflammation and cellular senescence have been found in various tissues or organs during aging or in age‐associated diseases (Ma, Sun, Li, et al., 2020; Wang et al., 2018; Zou et al., 2020). Altogether, our results suggest that INK128 extends the functions of rOvaries through suppressing the mTOR pathway and inflammation or immune response as well as remarkably improving mitochondria functions. The findings that mitochondria functions are enhanced and inflammation suppressed by INK128 may have general implications in extending functions of the regenerative tissues in aging organism following stem cell transplantation.

Maintaining quiescence of follicle reserve by INK128 also could be important for maintaining stem cell functions for longer time *in vivo*. Similarly, in somatic cell types, quiescence also is a common feature of many stem cell populations (van Velthoven & Rando, 2019). Age‐associated declines in stem cell function are characterized by metabolic changes and particularly mitochondria dysfunction (Ren et al., 2017). Quiescent stem cells provide sources to enhance maintenance and repair of aged or diseased tissues (van Velthoven & Rando, 2019). Activation of quiescent postmitotic muscle stem cells and neural stem cell activation is involved in regeneration and aging (Adams et al., 2015; Leeman et al., 2018; de Morree et al., 2019; van Velthoven & Rando, 2019). Furthermore, the FOXO family of transcription factors, which inhibits oxidative and/or metabolic stress, is functionally important in quiescent stem cells to safeguard these cells from environmental stress (Cheung & Rando, 2013). This is consistent with the notion that FOXO3 promotes quiescence in adult muscle stem cells during the process of self‐renewal (Gopinath et al., 2014). In contrast, mTORC1 activity is necessary and sufficient for exit from quiescent state of stem cells (Rodgers et al., 2014). Our data reveal that INK128 effectively activates and elevates FOXO3 levels in the germ cell‐reconstructed ovaries. The quiescent state prevents the premature activation or differentiation of stem cells. Exhaustion of the stem cell pool results in impaired tissue homeostasis and regeneration, highlighting the importance of maintaining stem cell quiescence for tissue and organismal health (Cheung & Rando, 2013). Chronic mTORC1 activation leads to stem cell loss, and somatic stem cells transiently activate mTORC1 signaling during tissue regeneration (Haller et al., 2017). TSC/mTORC1 signaling has an important regulatory role in stem cell maintenance and differentiation. It will be interesting to test whether mTOR inhibition by INK128 can delay activation and thus aging of quiescent somatic stem cells.

In C. elegans, the prevention of germline stem cell proliferation results in a 60% extension of life span, termed gonadal longevity (Antebi, 2013). Gonadal longevity relies on the transcriptional activities of steroid nuclear receptor DAF‐12, the FOXO transcription factor homolog DAF‐16, the FOXA transcription factor homolog PHA‐4, and the HNF‐4‐like nuclear receptor NHR‐80. Because the reproductive system also regulates longevity in other species (Antebi, 2013), INK128 may also benefit organismal longevity, in addition to prolonged reproductive and endocrine functions.

PGC‐like cells (PGCLCs) have been successfully achieved from pluripotent stem cells, embryonic stem cells, or induced pluripotent stem cells (Hayashi et al., 2012; Hikabe et al., 2016), as well as from chemically induced pluripotent stem cells by pure chemical reprogramming of adult somatic granulosa cells (Tian et al., 2019). PGCLCs reaggregated with E12.5 female gonadal somatic cells are subjected to ovarian cyst transplantation to obtain functional mature eggs (Hayashi et al., 2012). Excitingly, PGCLCs derived from granulosa cells also can reconstitute ovarian functions in the recipient mice following kidney capsule transplantation (Tian et al., 2019). It is likely that PGCLCs derived from pluripotent stem cells or reprogrammed from somatic cells could be used to extend ovarian functions in combination with mTOR inhibition by INK128, opening wider applications in preservation of fertility and endocrine functions.

## 4 EXPERIMENTAL PROCEDURES

### 4.1 Animal care and use

The use of mice for this research was approved by the Nankai University Animal Care and Use Committee. All mice used in this study were taken care of and operated according to the relevant regulations. Mice were housed and cared in individually ventilated cages (IVCs) on a standard 12‐h light: 12‐h dark cycle in the sterile Animal Facility. Three‐five‐month‐old second‐generation *Terc*‐deficient (G2 *Terc* ^–/–^) mice in C57BL/6 background, C57BL/6‐Tg (CAG‐EGFP) C14‐Y01‐FM131Osb mice that carry actin‐GFP obtained from Model Animal Research Center of Nanjing University. NOD SCID mice, C57BL/6NCrSlc (B6), and albino ICR mice purchased from Beijing Vital River Laboratory Animal Technology Co., Ltd, were used in this study.

### 4.2 Aggregation of PGCs

PGCs from C57BL/6‐GFP fetal ovaries were reaggregated with somatic cells from the same gonads. To obtain PGCs and somatic cells *in vivo*, female E12.5 gonads were collected from E12.5 embryos obtained by intercrosses of ICR or C57BL/6‐GFP mice. The mesonephros were surgically separated from the gonads using insulin syringe. Gonads were dissociated with 0.05% trypsin–EDTA (Invitrogen) by incubation at 37°C for 10 min, washed with DMEM+FBS medium, and collected by centrifugation. Large clumps of cells were removed using a cell strainer (BD Biosciences). The PGCs and somatic cells were plated in the wells of a low‐cell‐binding U‐bottom 96‐well Lipidure‐Coat plate in MF10 medium (MF10 medium contains M199 (Sigma) with 10% FBS, 1 mM l‐glutamine, and 1% 2A) added with 10 μM Rocki (modified by Ref. (Matoba & Ogura, 2011; Zeng et al., 2017)). The proportion of E12.5 PGCs and gonadal somatic cells was 1:6 per aggregation.

### 4.3 Transplantation of PGC aggregates into kidney capsule

Kidney capsule transplantation was performed based on the methods described (Qing et al., 2008; Sheng et al., 2019; Zeng et al., 2017). Briefly, one or two aggregates were implanted in the “pocket,” which was made between the kidney capsule and kidney tissue of a bilaterally ovariectomized (OE) recipient mouse. Transplantation procedure was completed in 5 min for each mouse. Eight‐ to 10‐month‐old (average of 36 weeks, reproductive aging) C57BL/6, 2‐ to 3–month‐old (average of 10 weeks) G2 *Terc* ^−/−^mice, 6‐ to 8‐week‐old NOD SCID, or young (2‐ to 3‐month‐old) C57BL/6 females were used as recipients of transplantation of PGCs. For hormone assay below, these recipients were randomly divided into two groups as follows: group 1: Each aggregate was transplanted beneath the kidney capsule of a bilaterally ovariectomized recipient mouse; group 2: A bilaterally ovariectomized recipient mouse without aggregates served as negative control. For transplantation into 36‐week‐old C57BL/6, 10‐week young G2 *Terc*−/− mice, or 10‐week young C57BL/6 recipient females, donor aggregates were PGCs and the somatic cells were isolated from E12.5 fetal gonads obtained by intercrosses of C57BL/6 females with C57BL/6‐actin‐GFP male mice. For transplantation into 6‐ to 8‐week NOD SCID recipient females, aggregates of PGCs and somatic cells from E12.5 fetal gonads of ICR mice intercrosses were used.

### 4.4 Rapamycin and INK128 administration

Rapamycin was obtained from LC Lab (Catalog No. R5000‐1). Purity of rapamycin, evaluated by HPLC, was >99%. Rapamycin was dissolved in drinking water at the concentration of 40 mg/L, and this concentration approximated 8.0 mg/kg/day for the treatment of young C57BL/6 mice. INK128 was obtained from Selleck.cn (MLN0128, Catalog No. S2811). Purity of INK128, evaluated by HPLC, was 99.68%. INK128 was dissolved in DMSO at stock concentration of 50 mM. Based on our preliminary experiments on treatment dosage, the recipient mice were treated with INK128 at the final concentration of 30 μM/L in drinking water, approximately 1.8 mg /kg body weight/day.

In our preliminary experiments, we tested the treatment concentration and time effects of rapamycin or INK128 on ovarian functions. Rapamycin at high concentration of 60 mg/L was not readily dissolved in water and resulted in some precipitates. While rapamycin at 30 mg/L gave rise to variable results, rapamycin at 40 mg/L produced consistent results and thus was used for subsequent experiments. We designed three different concentrations, 20, 30, and 40 μM/L, for INK128 preliminary experiments. By treatment of recipient mice with INK128 at 40 μM/L for 2 months, certain number of secondary follicles can be seen, but granulosa cells were heavily stained, follicle structure, and development appeared abnormal, showing some empty vacuoles (data not shown). These results imply that too high concentration of INK128 may affect normal follicle development. Based on the data also shown in Figure 1 and Figure S2, we chose INK128 at 30 μM/L, which achieved normal ovary‐like structure and follicles for subsequent transplant experiments.

### 4.5 Follicle count

The aggregate‐formed rOvaries were carefully retrieved and subsequently dehydrated with graded alcohols, cleared in xylene, and embedded in paraffin wax. The serial section (5 μm) from each rOvary was aligned in order on glass microscope slides, stained with hematoxylin–eosin (H&E), and analyzed for the number of follicles at various developmental stages in every fifth section with random start from the first five sections. The total number of follicles per rOvary was calculated by combining the counts of every fifth section throughout the whole rOvary, based on method described (Liu et al., 2013; Tilly, 2003). The follicles were categorized into primordial, primary, secondary, and antral or mature accordingly. Secondary follicles were characterized as having more than one layer of GCs with no visible antrum. Antral or mature follicles possessed small areas of follicular fluid (antrum) or a single large antral space (Myers et al., 2004). Only those follicles containing an oocyte with a clearly visible nucleus were scored.

### 4.6 Fluorescence microscopy of rOvary sections

Briefly, after being deparaffinized, rehydration, and wash in 0.01 M PBS (pH =7.2), sections were incubated with 3% H_2_O_2_ for 10 min at room temperature to block endogenous peroxidase, subjected to high‐pressure antigen recovery sequentially in 0.01 M citrate buffer (pH =6.0) for 3 min, permeabilized in 0.1% Triton X‐100 for 30 min, then washed once in PBS, and incubated with blocking solution (5% goat serum and 0.1% BSA in PBS) for 2 h at room temperature and then with the diluted primary antibodies overnight at 4°C. The following primary antibodies were used: Vasa (ab13840, Abcam, 1:200) and Foxl2 (ab5096, Abcam, 1:200). Blocking solution without the primary antibody served as negative control. After washing with PBS, sections were incubated with appropriate secondary antibodies, Alexa Fluor® 594 donkey anti‐goat IgG (H + L) or FITC goat anti‐rabbit IgG (H + L). Nuclei were stained with 0.5 μg/ml Hoechst in VECTASHIELD mounting medium. Fluorescence was detected and imaged using Axio Imager Z2 Fluorescence Motorized Microscope (Carl Zeiss).

### 4.7 Hormone assays

Serum follicle‐stimulating hormone (FSH), estradiol (E2), and anti‐Müllerian hormone (AMH) levels were assayed by ELISA Kit (CK‐E20381, CK‐E20419, and CK‐E90200; Hangzhou Eastbiopharm Co., Ltd). Quality control serum, sterilized distilled water, and five series diluted standard samples for a standard curve were tested for each serum sample. The intra‐ and inter‐assay coefficients of variability for AMH, FSH, and E2 were below 8% and 12%.

### 4.8 Western blot

rOvaries were washed twice in PBS, collected, ground, and lysed in cell lysis buffer on ice for 30 min and then sonicated for 1 min at 60 of amplitude at 2‐s intervals. After centrifugation at 13,600 rpm at 4 °C for 10 min, supernatant was transferred into new tubes. The concentration of the protein sample was measured by bicinchoninic acid, and then, protein samples were boiled in SDS sample buffer at 95 °C for 10 min. One microgram total protein of each cell extract was resolved by 10% Acr‐Bis SDS‐PAGE and transferred to polyvinylidene difluoride membranes (PVDF, Millipore). Nonspecific binding was blocked by incubation in 5% skim milk or 5% BSA in TBST at room temperature for 2 h. Blots were then probed by incubation overnight at 4°C with primary antibodies Foxo3 (CST, #2497S, 1:1000), P‐AKT (CST, #4060S, 1:1000), AKT (CST, #4685S, 1:1000), or β‐actin served as loading control. Immunoreactivity bands were then probed for 2 h at room temperature with the appropriate horseradish peroxidase (HRP)‐conjugated secondary antibodies, goat anti‐rabbit IgG‐HRP, or goat anti‐mouse IgG (H + L)/HRP. Protein bands were detected by chemiluminescent HRP substrate (WBKLS0500, Millipore).

### 4.9 Library preparation and RNA sequencing

A total amount of 3 g of RNA per sample was used as input material for the RNA preparation. Sequencing libraries were generated using NEBNext Ultra RNA Library Prep Kit for Illumina (NEB, United States) following manufacturer's recommendations, and index codes were added to attribute sequences to each sample. RNA‐seq raw reads with low‐quality bases, and adapters were trimmed by Trimmomatic (Bolger et al., 2014), to obtain clean reads. Clean reads were aligned to the UCSC mouse mm10 reference genome using the Hisat2 with default settings (Kim et al., 2015). Read counts of each gene annotated in RefGene were calculated by featureCounts (Liao et al., 2014). The read counts were loaded into RStudio (v4.0.1). To ensure the accuracy of estimated gene expression levels and subsequent analysis, only genes with normalized expression (CPM) >10 were analyzed. DESeq2 (Love et al., 2014) was used to obtain statistical significance of differentially expressed genes between INK128 treatment and control groups. Only the genes with a fold change of log2‐transformed larger than log_2_(1.5) and false discovery rate (FDR) less than 0.05 from DEseq2 results were considered to be differentially expressed. Gene ontology (GO) and KEGG analysis of differentially expressed genes were performed by DAVID (v 6.8) (Huang da et al., 2009), and only enrichment which showed *p* value < 0.05 were selected. The heat maps were drawn by the R package “pheatmap,” and the Venn diagram was drawn using the R package “venn.diagram.” The gene expression level was normalized by DESeq2.

### 4.10 Telomere measurement by quantitative PCR

Genome DNA was prepared using DNeasy Blood & Tissue Kit (Qiagen, Valencia, CA). PCRs were performed on the iCycler iQ5 2.0 Standard Edition Optical System (Bio‐Rad, Hercules, CA), using telomeric primers and primers for the reference control gene (mouse 36B4 single‐copy gene) (Callicott & Womack, 2006). For each PCR, a standard curve was made by serial dilutions of known amounts of DNA. The telomere signal was normalized to the signal from the single‐copy gene to generate a T/S ratio indicative of relative telomere length.

### 4.11 Statistical analysis

Data are represented as mean ±standard error of mean (SEM). Statistical significance was determined using the unpaired Student's t test of unequal variance. Differences were considered to be statistically significant for *p* < 0.05 (*), *p* < 0.01 (**), or *p* < 0.001 (***).

## CONFLICT OF INTEREST

The authors declare no competing interests.

## AUTHOR CONTRIBUTIONS

DH and XS performed major experiments and analyzed the data; CLT helped experiments; JL, LLL, and MG performed RNA‐seq and data analysis; DH and XS prepared the manuscript; all authors reviewed and edited the manuscript; and LL conceived the project and revised the manuscript.

## Supporting information

Fig S1‐S4

## Data Availability

RNA‐sequencing data have been deposited in the GEO database (GSE157325). Other data are available upon request.
