# Supplementary material for: Mtor inhibition by INK128 extends functions of the ovary reconstituted from germline stem cells in aging and premature aging mice
Source: Aging Cell. 2021 Jan 14;20(2):e13304. doi: 10.1111/acel.13304 (PMC7884035; doi:10.1111/acel.13304)
Supplement: Supplementary file 1 — Fig S1‐S4 [file ACEL-20-e13304-s001.docx]

Supporting information

Figures S1-4


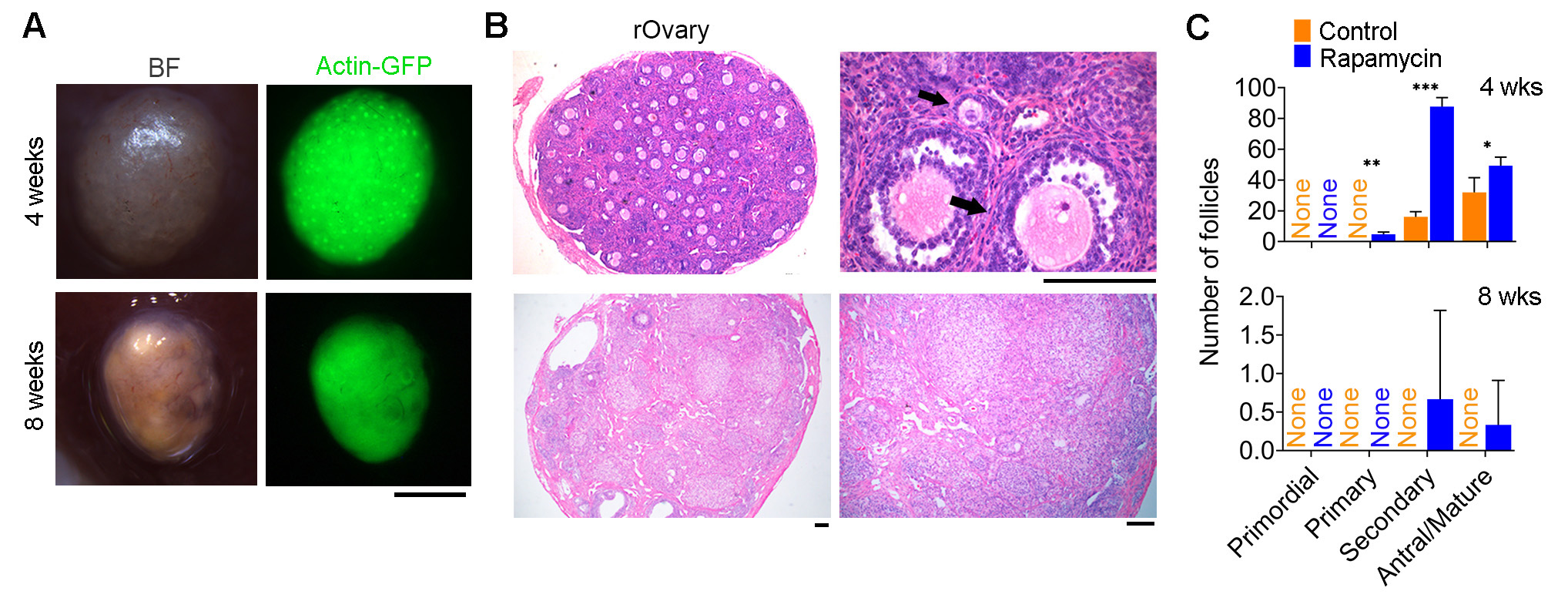


Figure S1. Follicular development of the reconstituted ovaries (rOvary) 4 or 8 weeks following transplantation of PGCs into young recipient C57BL/6 mice (2-3 months old) treated with or without rapamycin.

(A) Morphology of the transplant/rOvary. Mice were treated with rapamycin at 40 mg/L in the water. After 4 weeks of transplantation, the follicle development in ovarian-like grafts can be visually observed through GFP fluorescence. Scale bar = 1 mm.

(B) Section histology by H&E staining of the rOvary, showing many follicles after 4 weeks following transplantation in the recipient mice treated with rapamycin, mainly secondary and antral follicles but almost no follicles present at eight weeks following transplantation. Black arrows indicate primary follicles and antral follicles. Control, recipient mice without rapamycin treatment. Scale bar = 100 μm.

(C) Number of follicles at various developmental stages 4 or 8 weeks after transplantation of PGCs in rapamycin treated recipient mice compared with controls without rapamycin. Mean ± SEM. n=3. * P <0.05, ** P<0.01, ***P<0.001.


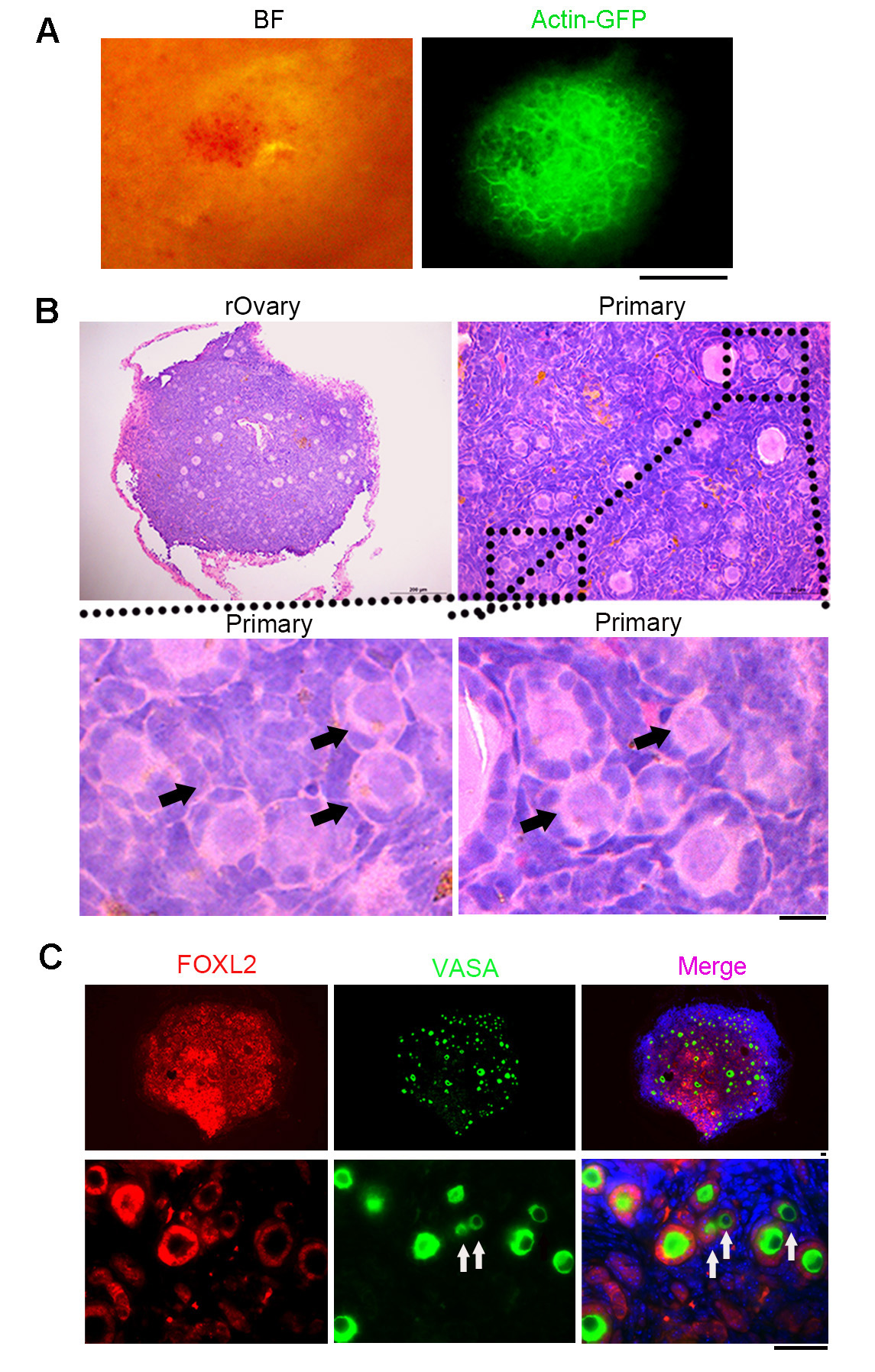


Figure S2. INK128 prevents early activation of folliculogenesis in the reconstituted ovaries (rOvary) of young C57BL/6 recipient mice.

(A) Morphology of reconstituted ovaries (rOvary) 4 weeks following transplantation into young recipient C57BL/6 mice (2-3 months old) of PGCs aggregated with E12.5 gonadal somatic cells. BF, microscopic image under bright field; Actin-GFP indicates donor cell sources from Actin-GFP mice. Scale bar = 500 μm.

(B) Histology of rOvary sections by H&E staining indicating various follicles four weeks following transplantation. The black arrows refer to primordial/primary follicles. Scale bar = 10 μm at higher magnification.

(C) Immunofluorescence of FOXL2 indicating granulosa cells in the follicles and VASA to label oocytes. Scale bar = 20 μm.


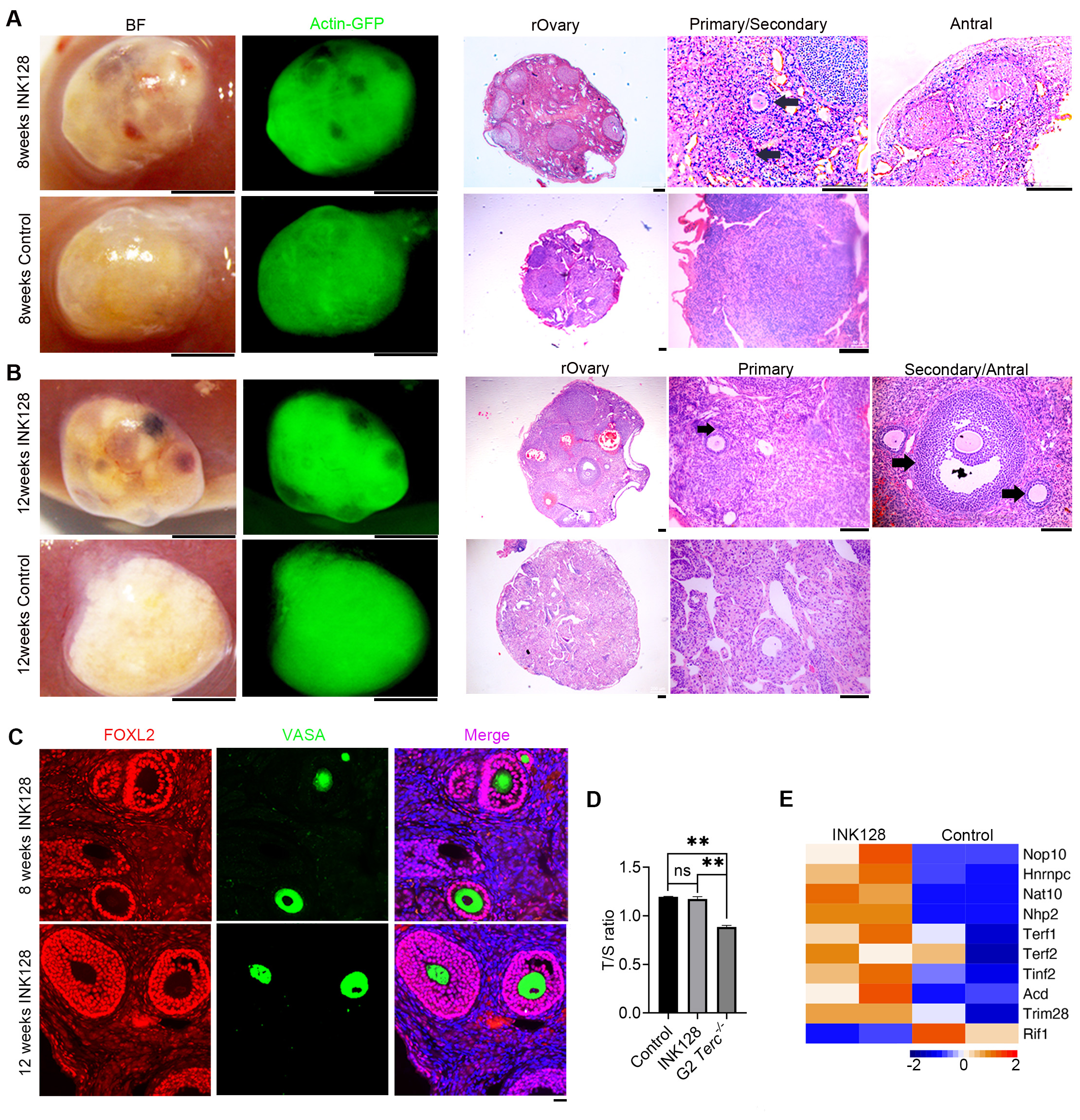


Figure S3. Follicle development in the rOvary of G2 *Terc*^-/-^ mice treated with or without INK128 for 8 or 12 weeks.

(A) Morphology of the rOvary and histology of rOvary sections by H&E staining 8 weeks following transplantation of PGCs aggregates in G2 *Terc*^-/-^ mice. BF, microscopic image under bright field; Actin-GFP indicates donor cell sources from Actin-GFP mice. scale bar = 1 mm. Black arrows indicate primary, secondary and mature follicles. scale bar = 100 μm. Young G2 *Terc*^-/-^ mice at the age of 10 weeks old were received the transplants.

(B) Morphology of the rOvary and histology of rOvary sections by H&E staining 12 weeks following transplantation of PGCs aggregates in G2 *Terc*^-/-^ mice. Young G2 *Terc*^-/-^ mice at the age of 10 weeks old were received the transplants.

(C) Immunofluorescence of FOXL2 indicating granulosa cells in the follicles and VASA to label oocytes. Scale bar = 20 μm.

(D) Relative telomere length shown as T/S ratio of rOvary from G2 *Terc^-/-^* recipient mice treated with or without INK128 (Control) for 4 weeks and ovary of 10 weeks-old G2 *Terc^-/-^*  mice. Mean ± SEM, n>=2; **P<0.01.

(E) Expression levels of genes associated with telomere maintenance and stabilization in rOvaries of INK128-treated and control mice.


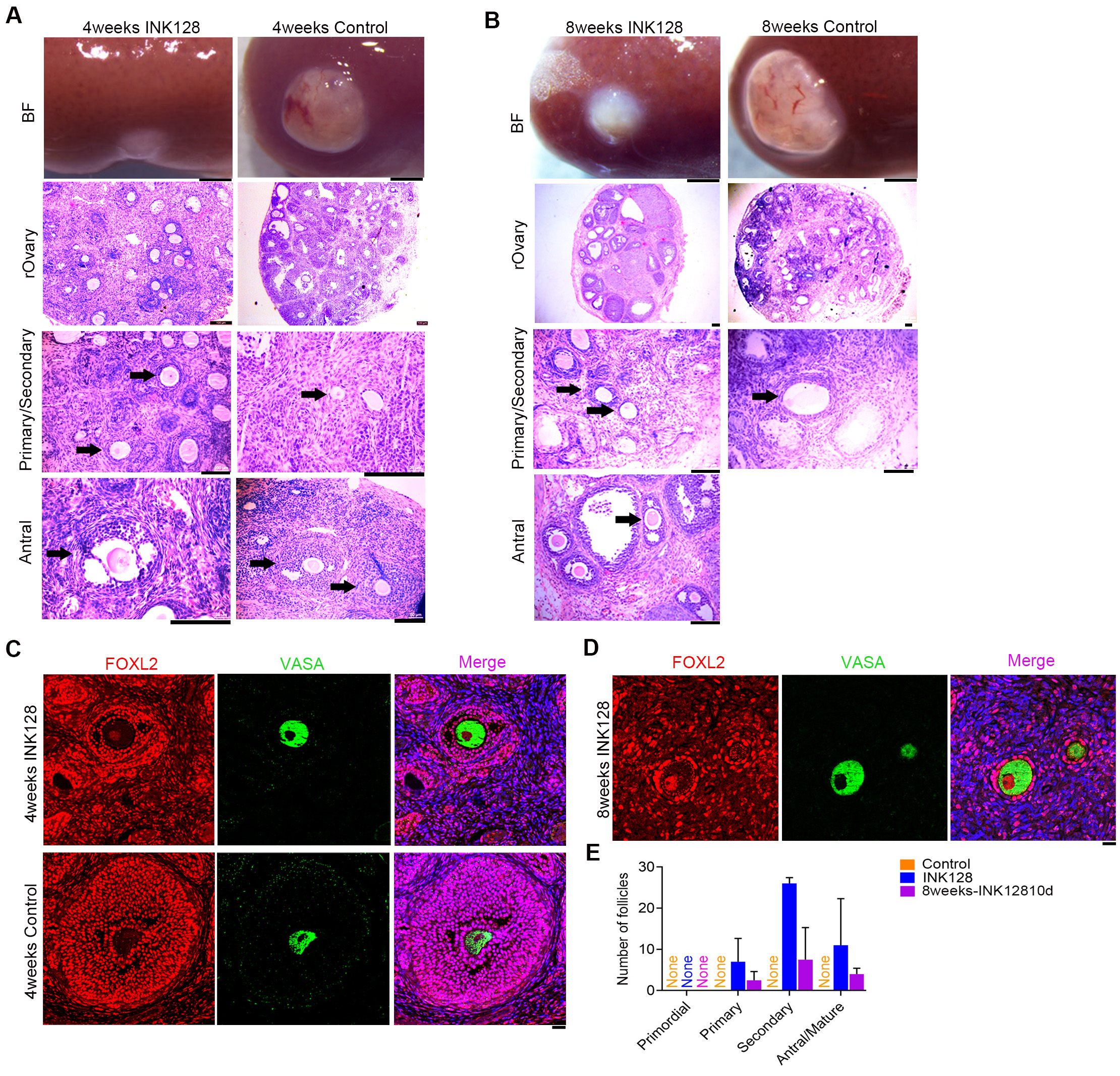
Figure S4. Follicular development in the rOvary of NOD SCID recipient mice treated with or without INK128 following transplantation of PGCs.

(A) Morphology under bright field and histology by H&E staining in sections of rOvary following treatment with INK128 for four weeks, compared with those of controls without receiving INK128. Black arrows indicate follicles at various developmental stages. Scale bar = 1 mm under bright field microscopy. Scale bar = 100 μm for histology.

(B) Morphology under bright field and histology by H&E staining in sections of rOvary following treatment with INK128 for eight weeks, compared with those of controls without receiving INK128. Black arrows indicate follicles at various developmental stages. Scale bar = 1 mm under bright field microscopy. Scale bar = 100 μm for histology.

(C,D) Immunofluorescence of germ cell marker VASA and granulosa cell marker FOXL2. Scale bar = 20 μm.

(E) Number of follicles in the rOvary eight weeks following transplantation. Control, recipient mice were not treated with INK128; INK128, recipient mice treated with INK128 for 8 weeks; 8 weeks-INK128 10d, recipient mice were treated with INK128 for 8 weeks, then removed from INK128 treatment for 10 days.
